# Abstract generalized vector quasi-equilibrium problems in noncompact Hadamard manifolds

**DOI:** 10.1186/s13660-017-1375-2

**Published:** 2017-05-10

**Authors:** Haishu Lu, Zhihua Wang

**Affiliations:** School of Business, Jiangsu University of Technology, Changzhou, Jiangsu 213001 P.R. China

**Keywords:** 90C33, 91E10, 65K05, 47J25, Hadamard manifold, maximal element, abstract generalized vector, quasi-equilibrium problem, variational inequality

## Abstract

This paper deals with the abstract generalized vector quasi-equilibrium problem in noncompact Hadamard manifolds. We prove the existence of solutions to the abstract generalized vector quasi-equilibrium problem under suitable conditions and provide applications to an abstract vector quasi-equilibrium problem, a generalized scalar equilibrium problem, a scalar equilibrium problem, and a perturbed saddle point problem. Finally, as an application of the existence of solutions to the generalized scalar equilibrium problem, we obtain a weakly mixed variational inequality and two mixed variational inequalities. The results presented in this paper unify and generalize many known results in the literature.

## Introduction

Let *K* be a nonempty subset of a nonempty set *X* and $f:X\times X \rightarrow\mathbb{R}$ be a bifunction. The scalar equilibrium problem (for short, SEP) is to find $\widehat{x}\in K$ such that $f( \widehat{x},y)\geq0$ for every $y\in K$. It is well known that SEP contains a broad class of problems arising in pure and applied mathematics, such as fixed point, minimax and variational inequality, Nash equilibrium, complementarity, and convex optimization problems (see, for example, [1–5] and the references therein). Recently, in a linear setting, many authors focused on the existence of solutions to equilibrium problems for vector mappings; see, for example, Ansari and Yao [6], Ansari and Flores-Bazán [7], Fu [8], Fu and Wan [9], Khan [10], Khan et al. [11], Khan and Chen [12], Hou et al. [13], Iusem and Sosa [14], Chen et al. [15], Kassay et al. [16], and the authors referenced by their works.

On the other hand, Riemannian manifolds provide a useful framework for the research of the related problems in optimization and equilibrium. Actually, many concepts and techniques fitting in Euclidean spaces have been extended to Riemannian manifolds. Most of the generalized methods require the sectional curvature of Riemannian manifold to be nonpositive. In fact, a large class of Riemannian manifolds, including Hadamard manifolds, possesses this important property which implies tight topological restrictions and rigidity phenomena (see [17]). Hadamard manifolds have turned out to be a suitable framework for diverse disciplines (see, for example, [18–20]). Applying the KKM principle allowed Colao et al. [21] to confirm the existence of solutions to the SEP in Hadamard manifolds. Approximately at the same time, Yang and Pu [22] studied the existence and stability of solutions to the SEP in Hadamard manifolds. By applying the KKM principle, Zhou and Huang [23] proved the existence of solutions to the vector optimization problem in Hadamard manifolds. Similarly, Li and Huang [24] presented some existence results of solutions to the generalized vector quasi-equilibrium problems in Hadamard manifolds. Recently, Batista et al. [25] introduced and studied the generalized vector equilibrium problem in Hadamard manifolds. Their results generalize the corresponding results of Colao et al. [21], Zhou and Huang [23], and Németh [26].

Motivated by the recent work mentioned above, the main purpose of this paper is to introduce and study the abstract generalized vector quasi-equilibrium problem (for short, AGVQEP) in noncompact Hadamard manifolds. The rest of this paper is organized as follows. In Section 2, we introduce notation, definitions, and preliminary results used in the paper. In Section 3, we apply an existence result of maximal elements in noncompact Hadamard manifolds in order to prove an existence theorem of solutions to AGVQEP under some suitable conditions. Applications to an abstract vector quasi-equilibrium problem (for short, AVQEP), a generalized scalar equilibrium problem (for short, GSEP), SEP, and a perturbed saddle point problem are provided. Section 4 is devoted to investigating the weakly mixed variational inequality problem (for short, WMVIP) in noncompact Hadamard manifolds. Our methods are based on a result concerning the existence of solutions to GSEP. Conclusions are presented in Section 5.

## Preliminaries

In this section, we recall some notation, definitions, and auxiliary results, which are intended to be used throughout this paper. These can be found in [27–33].

Let $\mathbb{R}$ denote the set of all real numbers. Let *X* be a set. Then we let ${\mathcal {F}}(X)$ represent the family of nonempty finite subsets of *X*. Let *A* be a subset of a topological space *X*, and then let int*A* and cl*A* denote the interior of *A* in *X* and the closure of *A* in *X*, respectively. Moreover, if $A\subseteq B\subseteq X$, then $\operatorname{int}_{B}A$ (respectively, $\operatorname{cl}_{B}A$) stands for the interior (respectively, closure) with respect to the topology of *B*, induced by that of *X*. Given two nonempty sets *X*, *Y* and a set-valued mapping $T:X\rightrightarrows Y$, the inverse $T^{-1}:Y\rightrightarrows X$ of *T* is defined by $T^{-1}(y)=\{x\in X:y\in T(x)\}$ for every $y\in Y$. Let *X* and *Y* be two topological spaces. Then a set-valued mapping $T:X\rightrightarrows Y$ is said to be upper semicontinuous (respectively, lower semicontinuous) on *X* iff, for each $x\in X$ and each open set $V\subseteq Y$ with $T(x)\subseteq V$ (respectively, $T(x)\cap V \neq\emptyset$), there exists an open neighborhood $U(x)$ of *x* in *X* such that $T(z)\subseteq V$ (respectively, $T(z)\cap V\neq\emptyset $) for every $z\in U(x)$. Let $\delta_{n}$ be the standard *n*-dimensional simplex with vertices $\{e_{0},e_{1},\ldots,e_{n}\}$ and for each $I\subseteq\{0,1,\ldots,n\}$, let $\delta_{\vert I\vert -1}$ denote the simplex with vertices $\{e_{j}:j\in I\}$, where $\vert I\vert $ denotes the cardinality of *I*.

Let *E* be a simply-connected *m*-dimensional manifold. For each $x\in E$, we denote by $T_{x}E$ the tangent space of *E* at *x*. Let $TE=\bigcup_{x\in E}T_{x}E$ denote the tangent bundle of *E*, which is naturally a manifold. A vector field on *E* is a mapping $\sigma:E \rightarrow TE$ such that $\sigma(x)\in T_{x}E$ for every $x\in E$. Let $\langle\cdot,\cdot\rangle_{x}$ denote the scalar product on $T_{x}E$ with the associated norm $\Vert \cdot \Vert _{x}$, where the subscript *x* will be omitted in the sequel when no confusion arises. If *E* is a differentiable manifold equipped with a scalar product $\langle\cdot,\cdot\rangle$ that varies smoothly from point to point, then we say that *E* is a Riemannian manifold. The family $\langle\cdot,\cdot\rangle$ of scalar products is called a Riemannian metric. We always assume that *E* can be endowed with a Riemannian metric to become a Riemannian manifold. Given a piecewise smooth curve $\gamma:[a,b]\rightarrow E$ joining *x* to *y* (i.e., $\gamma(a)=x$ and $\gamma(b)=y$), we can define the length of *γ* as follows:

$$A(\gamma)= \int_{a}^{b} \bigl\Vert \gamma^{\prime}(t) \bigr\Vert \,dt. $$

Then, for any two points $x, y\in E$, the Riemannian distance $d(x,y)$ is defined by

$$d(x,y)=\inf \bigl\{ A(\gamma):\gamma \mbox{ is a piecewise smooth curve joining } x \mbox{ and } y \bigr\} . $$

A vector field *σ* is said to be parallel along *γ* if $\nabla_{\gamma^{\prime}}\sigma=0$, where ∇ is the Levi-Civita connection associated with $(E,\langle\cdot,\cdot\rangle)$ and *γ* is a smooth curve in *E*. If $\gamma^{\prime}$ itself is parallel along *γ*, then *γ* is called a geodesic, and in this case, $\Vert \gamma^{\prime} \Vert $ is constant. When $\Vert \gamma ^{\prime} \Vert =1$, *γ* is said to be normalized. A geodesic which joins *x* to *y* in *E* is said to be minimal if its length equals $d(x,y)$.

A Riemannian manifold *E* is geodesically complete if, for each $x\in E$, all geodesics starting from *x* are defined for every $t\in\mathbb{R}$. By the Hopf-Rinow theorem (see [31]), we can see that if a Riemannian manifold is geodesically complete, then $(E,d)$ is a complete metric space and each bounded closed subset is compact. In addition, for any two points in *E*, there exists a minimal geodesic joining these two points.

A Hadamard manifold *E* is a complete simply connected Riemannian manifold of nonpositive sectional curvature. Unless explicitly stated otherwise, throughout the remainder of this paper, we assume that *E* is a finite dimensional Hadamard manifold and *V* is a Hausdorff topological vector space.

### Definition 2.1

[31]

Let $x\in E$. The exponential mapping $\operatorname{exp}_{x}:T_{x}E\rightarrow E$ at *x* is defined by $\operatorname{exp}_{x}v=\gamma_{v}(1,x)$ for every $v\in T_{x}E$, where $\gamma_{v}$ is the geodesic starting at *x* with velocity *v* (i.e., $\gamma(0)=x$, $\gamma^{\prime}(0)=v$).

### Lemma 2.1

[33]

*Let* $x\in E$. *Then* $\operatorname{exp}_{x}:T _{x}E\rightarrow E$ *is a diffeomorphism*, *and for any two points* $x, y\in E$, *there exists a unique minimal normalized geodesic* $\gamma_{x,y}=\operatorname{exp}_{x}t\operatorname{exp}_{x}^{-1}y$ *for every* $t\in[0,1]$ *joining* *x* *to* *y*.

So from now on, a geodesic means the unique minimal normalized one.

### Definition 2.2

[23]

A set $C\subseteq E$ is said to be convex iff, for any two points $x, y\in C$, the geodesic joining *x* to *y* is contained in *C*; that is, $\gamma_{x,y}=\operatorname{exp}_{x}t \operatorname{exp}_{x}^{-1}y\in C$ for every $t\in[0,1]$.

### Definition 2.3

[23]

A real-valued function $f:E\rightarrow \mathbb{R}$ is said to be convex iff, for any two points $x, y\in E$, we have $f(\operatorname{exp}_{x}t\operatorname{exp}_{x}^{-1}y)\leq tf(x)+(1-t)f(y)$ for every $t\in[0,1]$. *f* is said to be concave iff −*f* is convex.

### Definition 2.4

Let *Q* be a nonempty subset of *V*. The set *Q* is called a convex cone iff $Q+Q\subseteq Q$ and $\lambda Q\subseteq Q$ for $\lambda\geq0$.

### Definition 2.5

Let $Q\subseteq V$ be a nonempty convex cone. A set-valued mapping $F:E\rightrightarrows V$ is said to be convex with respect to *Q* iff, for each $x, y\in E$ and each $t\in[0,1]$, we have $F(\operatorname{exp}_{x}t\operatorname{exp}_{x}^{-1}y)\subseteq tF(x)+(1-t)F(y)+Q$.

### Remark 2.1

Definition 2.5 includes Definition 2.3 as a special case. In fact, when *F* is a single-valued mapping, $V=\mathbb{R}$, and $Q=(-\infty,0]$, Definition 2.5 coincides with Definition 2.3. Also Definition 2.5 generalizes Definition 3.1 of Zhou and Huang [23] in the following aspects: (1) from a single-valued mapping to a set-valued mapping; (2) from the convex cone in Euclidean spaces to the convex cone in Hausdorff topological vector spaces.

### Definition 2.6

Let $Q\subseteq V$ be a nonempty convex cone. A set-valued mapping $F:E\rightrightarrows V$ is said to be quasiconvex-like with respect to *Q* iff, for each $x, y\in E$ and each $t\in[0,1]$, we have $F(\operatorname{exp}_{x}t\operatorname{exp}_{x}^{-1}y)\subseteq F(x)+Q$ or $F(\operatorname{exp}_{x}t\operatorname{exp}_{x}^{-1}y)\subseteq F(y)+Q$.

### Definition 2.7

[21]

Let $A\subseteq E$. The convex hull of *A* is defined by the smallest convex subset of *E* containing *A* and denoted by $\operatorname{conv}(A)$.

### Definition 2.8

Let *X* be a topological space. A set-valued mapping $F:X\rightrightarrows E$ is called a Fan-Browder mapping iff the following conditions are fulfilled:

(i) for each $x\in X$, $F(x)$ is nonempty and convex;
(ii) for each $y\in E$, $F^{-1}(y)$ is open in *X*.

### Definition 2.9

Let $G:E\rightrightarrows E$ be a set-valued mapping. Then *G* is said to be of class ${\mathcal {H}}_{E}$ iff the following conditions are satisfied:

- for each $x\in E$, $G(x)$ is convex;
- for each $x\in E$, $x\notin G(x)$;
- there exists a set-valued mapping $F:E\rightrightarrows E$ such that
  1. for each $x\in E$, $F(x)\subseteq G(x)$;
  2. $\bigcup_{y\in E}F^{-1}(y)=\bigcup_{y\in E}\operatorname{int}F^{-1}(y)$;
  3. $\{x\in E:F(x)\neq\emptyset\}=\{x\in E:G(x)\neq\emptyset\}$.

### Lemma 2.2

[34]

*Let* $x_{0}\in E$ *and* $\{x_{n}\}\subseteq E$ *such that* $x_{n}\rightarrow x_{0}$. *Then the following statements hold*:

(i) *for each* $y\in E$, $\operatorname{exp}_{x_{n}}^{-1}y\rightarrow \operatorname{exp}_{x_{0}}^{-1}y$ *and* $\operatorname{exp}_{y}^{-1}x_{n}\rightarrow \operatorname{exp}_{y}^{-1}x_{0}$;
(ii) *if* $\{v_{n}\}$ *is a sequence such that* $v_{n}\in T_{x _{n}}E$ *and* $v_{n}\rightarrow v_{0}$, *then* $v_{0}\in T_{x_{0}}E$;
(iii) *given the sequences* $\{u_{n}\}, \{v_{n}\}\subseteq T _{x_{n}}E$, *if* $u_{n}\rightarrow u_{0}$, $v_{n}\rightarrow v_{0}$ *with* $u_{0}, v_{0}\in T_{x_{0}}E$, *then* $\langle u_{n},v_{n}\rangle \rightarrow\langle u_{0},v_{0}\rangle$.

### Lemma 2.3

[35]

*Let* $x\in E$. *Then the following statements are equivalent*:

(i) *the function* $E\ni y\mapsto\langle u,\operatorname{exp}_{x}^{-1}y \rangle$ *is affine for every* $u\in T_{x}E$;
(ii) *the mapping* $\operatorname{exp}_{x}:T_{x}E\rightarrow E$ *is a global isometry*;
(iii) *for each* $q_{1}, q_{2}\in E$, *the curve* $[0,1] \ni t\mapsto \operatorname{exp}_{x}((1-t)\operatorname{exp}_{x}^{-1}(q_{1})+t \operatorname{exp}_{x}^{-1}(q_{2}))$ *is the minimal geodesic joining* $q_{1}$ *to* $q_{2}$;
(iv) *the sectional curvature of* *E* *is identically zero* (*i*.*e*., *E* *is isometric to the usual Euclidean space*).

Let $f:E\rightarrow\mathbb{R}$ be a real-valued function. The subdifferential of *f* is the set-valued mapping $\partial f:E\rightrightarrows TE$ defined by $\partial f(x)=\{u\in T_{x}E:\langle u,\operatorname{exp}_{x} ^{-1}y\rangle\leq f(y)-f(x), \forall y\in E\}$ for every $x\in E$ and its elements are called subgradients. For each $x\in E$, the subdifferential $\partial f(x)$ is a closed convex subset (possibly empty) of $T_{x}E$. Let $D(\partial f)=\{x\in E:\partial f(x)\neq \emptyset\}$ denote the domain of *∂f*.

The following lemma guarantees the existence of subgradients for convex functions.

### Lemma 2.4

[36]

*Let* $f:E\rightarrow\mathbb{R}$ *be a convex function*. *Then*, *for any* $x\in E$, *the subdifferential* $\partial f(x)$ *is a nonempty subset of* $T_{x}E$; *i*.*e*., $D(\partial f)=E$.

The following lemma, which provides the existence of maximal elements for a set-valued mapping, plays a key role in the proof of the existence of solutions to AGVQEP.

### Lemma 2.5

*Let* $G:E\rightrightarrows E$ *be of class* ${\mathcal {H}}_{E}$ *and* *K* *be a nonempty compact subset of* *E*. *Suppose that one of the following conditions holds*:

(i)_1_: *for each* $N\in{ \mathcal {F}}(E)$, *there exists a nonempty compact convex subset* $E_{N}$ *of* *E* *containing* *N* *such that* $E_{N}\setminus K\subseteq\bigcup_{y\in E_{N}}\operatorname{int}_{E_{N}}(G ^{-1}(y)\cap E_{N})$;
(i)_2_: *there exists a point* $y_{0}\in E$ *such that* $\operatorname{cl}(E\setminus G^{-1}(y_{0}))\subseteq K$.

*Then there exists* $\widehat{x}\in K$ *such that* $G(\widehat{x})= \emptyset$.

### Proof

Since *G* is of class ${\mathcal {H}}_{E}$, it follows that

- for each $x\in E$, $G(x)$ is convex;
- for each $x\in E$, $x\notin G(x)$;
- there exists a set-valued mapping $F:E\rightrightarrows E$ such that
  1. for each $x\in E$, $F(x)\subseteq G(x)$;
  2. $\bigcup_{y\in E}F^{-1}(y)=\bigcup_{y\in E}\operatorname{int}F ^{-1}(y)$;
  3. $\{x\in E:F(x)\neq\emptyset\}=\{x\in E:G(x)\neq \emptyset\}$.

We prove Lemma 2.5 by considering the following two cases.

*Case I.* Suppose that (i)_1_ holds. We proceed by contradiction. If the conclusion of Lemma 2.5 is false, then $G(x)\neq\emptyset$ for every $x\in K$. By (3), we have $F(x)\neq\emptyset$ for every $x\in K$, which implies that $K\subseteq\bigcup_{y\in E}F^{-1}(y)$. Moreover, it follows from (1) and (2) that $K\subseteq\bigcup_{y\in E}\operatorname{int}F^{-1}(y)\subseteq \bigcup_{y\in E}\operatorname{int}G^{-1}(y)$. Because *K* is a nonempty compact subset of *E*, we may choose $N\in{ \mathcal {F}}(E)$ such that

2.1 $$ K\subseteq\bigcup_{y\in N}\operatorname{int}G^{-1}(y). $$

By (i)_1_, there exists a nonempty compact convex subset $E_{N}$ of *E* containing *N* such that

2.2 $$ E_{N}\setminus K\subseteq\bigcup_{y\in E_{N}} \operatorname{int}_{E_{N}} \bigl(G ^{-1}(y)\cap E_{N} \bigr). $$

By (2.1), we get $K\cap E_{N}\subseteq\bigcup_{y\in N}\operatorname{int}_{E_{N}}(G^{-1}(y)\cap E_{N})$. Together with (2.2), we have $E_{N}=(E_{N}\setminus K)\cup(K\cap E_{N})= \bigcup_{y\in E _{N}}\operatorname{int}_{E_{N}}(G^{-1}(y)\cap E_{N})$. Applying the compactness of $E_{N}$, there exists $\{y_{0},y_{1},\ldots,y_{n}\} \in{ \mathcal {F}}(E_{N})$ such that $\{\operatorname{int}_{E_{N}}(G^{-1}(y_{i}) \cap E_{N})\}_{i=0}^{n}$ is a finite open cover of $E_{N}$. By Theorem 5.1 in [37], there is, for every *i*, a continuous function $\beta_{i}:E_{N}\rightarrow[0,1]$ such that $\sum_{i=0}^{n}\beta_{i}(x)=1$ for every $x\in E_{N}$ and such that $\beta_{i}(x)> 0$ implies $x\in \operatorname{int}_{E_{N}}(G^{-1}(y_{i}) \cap E_{N})$. Consequently, we can define a continuous mapping $f:E_{N}\rightarrow\delta_{n}$ by $f(x)=\sum_{i=0}^{n}\beta_{i}(x)e _{i}$ for every $x\in E_{N}$ with $f(x)=\sum_{i\in I(x)}\beta_{i}(x)e _{i}\in\delta_{\vert I(x)\vert -1}$, where $I(x)$ is defined by

$$i\in I(x)\quad \Leftrightarrow\quad \beta_{i}(x)>0\quad \Rightarrow \quad x\in \operatorname{int}_{E_{N}} \bigl(G^{-1}(y_{i})\cap E_{N} \bigr)\quad \Rightarrow\quad y_{i}\in G(x). $$

Hence, it follows from the convexity of $G(x)$ that $\operatorname{conv}(\{y _{i}:i\in I(x)\})\subseteq G(x)$.

Following the same argument as in the proof of Lemma 3.1 in [21], we can conclude that there exists a continuous mapping $\xi:\delta _{n}\rightarrow D(\{y_{0},y_{1},\ldots,y_{n}\})$ satisfying

$$\xi(\delta_{\vert I\vert -1})\subseteq D \bigl(\{y_{i}:i\in I\} \bigr), \quad \forall I \subseteq\{0,1,\ldots,n\}, $$

where $D(\{y_{0},y_{1},\ldots,y_{n}\}):=\bigcup_{i=0}^{n}D_{i}$, $D_{0}=\{y_{0}\}$ and, for each $i\in\{1,2,\ldots,n\}$, $D_{i}$ is defined by $D_{i}=\{z\in\gamma_{y_{0},x}:x\in D_{i-1}\}$. Then $D(\{y_{0},y_{1},\ldots,y_{n}\})$ is a closed subset of $\operatorname{conv}( \{y_{0},y_{1},\ldots,y_{n}\})$.

Now, the mapping $g=\xi\circ f$ has the property that $g(x)=\xi(f(x))\in\xi(\delta_{\vert I(x)\vert -1})\subseteq D(\{y_{i}:i \in I(x)\})\subseteq \operatorname{conv}(\{y_{i}:i\in I(x)\})\subseteq G(x)$ for every $x\in E_{N}$. Since the mapping $f\circ\xi:\delta_{n}\rightarrow \delta_{n}$ is continuous, it follows from the Brouwer fixed point theorem that there exists $p^{*}\in\delta_{n}$ such that $p^{*}=f( \xi(p^{*}))$. Let $\overline{x}=\xi(p^{*})$. Then we have $\overline{x}=\xi(p^{*})=\xi(f(\xi(p^{*})))=\xi(f(\overline{x})) \in G(\overline{x})$, which contradicts (b). Therefore, there must exist $\widehat{x}\in K$ such that $G(\widehat{x})=\emptyset$. This completes the proof.

*Case II.* Assume that (i)_2_ holds. Suppose, by way of contradiction, that $G(x)\neq \emptyset$ for every $x\in K$. By (3), we have $F(x)\neq\emptyset$ for every $x\in K$. Thus, by (1) and (2), we have $K\subseteq \bigcup_{y\in E}F^{-1}(y)=\bigcup_{y\in E}\operatorname{int}F^{-1}(y)\subseteq \bigcup_{y\in E}\operatorname{int}G^{-1}(y)$. Since *K* is compact, there exists $N_{0}\in{ \mathcal {F}}(E)$ such that

2.3 $$ K\subseteq\bigcup_{y\in N_{0}}\operatorname{int}G^{-1}(y). $$

By (i)_2_, we have $E\setminus K\subseteq \operatorname{int}G^{-1}(y_{0})$. Together with (2.3), we get

$$E=(E\setminus K)\cup K=\bigcup_{y\in N} \operatorname{int}G^{-1}(y), $$

where $N=\{y_{0}\}\cup N_{0}=\{y_{0},y_{1},\ldots,y_{n}\}\in {\mathcal {F}}(E)$. This implies that $\{\operatorname{int}G^{-1}(y_{i})\}_{i=0} ^{n}$ is a finite open cover of *E*. Since *E* is a normal space, it follows that there exists a finite collection of functions $\{\beta _{i}\}_{i=0}^{n}$ subordinated to the open cover $\{\operatorname{int}G^{-1}(y _{i})\}_{i=0}^{n}$, which implies that

$$\begin{aligned} \textstyle\begin{cases} \beta_{i}:E\rightarrow[0,1]\quad \mbox{is continuous for every } i \in\{0,1,\ldots,n\}; \\ \beta_{i}(x)>0\Rightarrow x\in \operatorname{int}G^{-1}(y_{i}); \\ \sum_{i=0}^{n}\beta_{i}(x)=1\quad \mbox{for every } x\in E. \end{cases}\displaystyle \end{aligned}$$

Consequently, the function on *E* defined by $f(x)=\sum_{i=0}^{n}\beta _{i}(x)e_{i}$ for every $x\in E$ with $f(x)=\sum_{i\in I(x)}\beta_{i}(x)e _{i}\in\delta_{\vert I(x)\vert -1}$ is continuous, where $I(x)$ is defined by

$$i\in I(x)\quad \Leftrightarrow\quad \beta_{i}(x)>0\quad \Rightarrow \quad x\in \operatorname{int}G ^{-1}(y_{i})\quad \Rightarrow\quad y_{i}\in G(x). $$

Hence, by the convexity of $G(x)$, we have $\operatorname{conv}(\{y_{i}:i \in I(x)\})\subseteq G(x)$.

By using the same method as in the proof of Lemma 3.1 in [21], we can conclude that there exists a continuous mapping $\xi:\delta _{n}\rightarrow D(\{y_{0},y_{1},\ldots,y_{n}\})$ satisfying

$$\xi(\delta_{\vert I\vert -1})\subseteq D \bigl(\{y_{i}:i\in I\} \bigr), \quad \forall I \subseteq\{0,1,\ldots,n\}, $$

where $D(\{y_{0},y_{1},\ldots,y_{n}\})$ is the same as in the previous case.

Now, let $g=\xi\circ f$. Then *g* has the following property: $g(x)\in\xi(\delta_{\vert I(x)\vert -1})\subseteq D(\{y_{i}:i\in I(x)\}) \subseteq \operatorname{conv}(\{y_{i}:i\in I(x)\})\subseteq G(x)$ for every $x\in E$. By the Brouwer fixed point theorem, the continuous mapping $f\circ\xi:\delta_{n}\rightarrow\delta_{n}$ has a fixed point $p^{*}\in\delta_{n}$; that is, $p^{*}=f(\xi(p^{*}))$. Let $\overline{x}=\xi(p^{*})$. Then we have $\overline{x}=\xi(p^{*})= \xi(f(\xi(p^{*})))=\xi(f(\overline{x}))\in G(\overline{x})$, which contradicts (b). Therefore, there must exist $\widehat{x}\in K$ such that $G(\widehat{x})=\emptyset$. This completes the proof. □

### Remark 2.2

Lemma 2.5 generalizes Theorem 3.1 of Yang and Pu [22] in the following aspects: (a) from compact Hadamard manifolds to noncompact Hadamard manifolds; (b) from one set-valued mapping to two set-valued mappings; (c) the condition that $\bigcup_{y\in E}F^{-1}(y)= \bigcup_{y\in E}\operatorname{int}F^{-1}(y)$ is weaker than (2) of Theorem 3.1 of Yang and Pu [22]; (d) concerns the more general Hadamard manifold with its sectional curvature being nonpositive instead of the Hadamard manifold with its sectional curvature being identically zero. This fact can be deduced from Lemma 2.3.

### Remark 2.3

(i)_1_ of Lemma 2.5 can be replaced by the following equivalent condition.

- For each $N\in{ \mathcal {F}}(E)$, there exists a nonempty compact convex subset $E_{N}$ of *E* containing *N* such that $E_{N}\setminus K\subseteq\bigcup_{y\in E_{N}}\operatorname{int}G^{-1}(y)$.

## Abstract generalized vector quasi-equilibrium problem

In this section, we introduce AGVQEP in Hadamard manifolds and present a sufficient condition for the existence of solutions to AGVQEP. As applications, we obtain results to solve AVQEP, GSEP, SEP, and the perturbed saddle point problem in noncompact Hadamard manifolds.

Let *K* be a nonempty subset of *E*, *W* be a nonempty set, and let $H:E\rightrightarrows E$, $C:E\rightrightarrows W$, $\psi:E \times E\rightrightarrows W$ be set-value mappings. We consider the following AGVQEP as follows: find $\widehat{x}\in K$ such that

$$\widehat{x}\in H(\widehat{x}) \quad \mbox{and}\quad \psi(\widehat{x},y) \nsubseteq C( \widehat{x}),\quad \forall y\in H(\widehat{x}). $$

It is worthwhile noting that AGVQEP is motivated by the generalized vector quasi-equilibrium problem introduced by Ansari and Flores-Bazán [7]. In particular, let $E=\mathbb{R}^{n}$, *W* be a Hausdorff topological vector space, and let $P:\mathbb{R} ^{n}\rightrightarrows W$ be a set-valued mapping such that, for each $x\in\mathbb{R}^{n}$, $P(x)$ is a closed and convex cone with $\operatorname{int}P(x)\neq\emptyset$. Moreover, let $C:\mathbb{R}^{n} \rightrightarrows W$ be a set-valued mapping defined by $C(x)=- \operatorname{int}P(x)$ for every $x\in\mathbb{R}^{n}$. Then AGVQEP retrieves a particular instance of the equilibrium problem in [7]. Here we would like to point out that the feasible set of AGVQEP is controlled by a set-valued mapping. In the real world, there are important problems which can be regarded as AGVQEPs in which the condition that the feasible set of AGVQEP is controlled by a set-valued mapping must be satisfied; for example, the equilibrium problems of the generalized games in Dasgupta and Maskin [38], Smeers et al. [39], Krawczyk [40], and Ansink and Houba [41].

### Remark 3.1

If $H(x)\equiv E$ for every $x\in E$, *W* is a Hausdorff topological vector space, and each $C(x)$ is replaced by $-\operatorname{int}C(x)$, where $C(x)$ is a closed and convex cone with $\operatorname{int}C(x)\neq\emptyset$, then AGVQEP reduces to the generalized vector equilibrium problem investigated by Batista et al. [25]. By the arguments in [25], we can see that AGVQEP also includes the equilibrium problems in [21, 23, 26] as its special cases.

### Remark 3.2

Some other special cases of AGVQEP are given as follows.

(I) Let $H(x)\equiv E$ for every $x\in E$. Then AGVQEP reduces to the abstract vector quasi-equilibrium problem (for short, AVQEP), which consists in finding $\widehat{x}\in E$ such that $\psi(\widehat{x},y) \nsubseteq C(\widehat{x})$ for every $y\in E$.
(II) If $W=\mathbb{R}$, $C(x)\equiv(-\infty,0)$ for every $x\in E$, and $F=f$, where $f:E\times E\rightarrow\mathbb{R}$ is a bifunction, then GVQEP reduces to the generalized scalar equilibrium problem (for short, GSEP), which is to find $\widehat{x}\in E$ such that $\widehat{x}\in H(\widehat{x})$ and $f(\widehat{x},y)\geq0$ for every $y\in H(\widehat{x})$. Furthermore, if $H(x)\equiv E$ for every $x\in E$, then GSEP reduces to SEP.

Now, we are ready, by using Lemma 2.5, to present the following existence theorem of solutions to AGVQEP in noncompact Hadamard manifolds.

### Theorem 3.1

*Let* $K\subseteq E$ *be a nonempty compact set and* *W* *be a nonempty set*. *Let* $\varsigma, \psi:E\times E\rightrightarrows W$, $C:E\rightrightarrows W$ *be three set*-*valued mappings and* $H:E\rightrightarrows E$ *be a Fan*-*Browder mapping*. *Assume that*

(i): *the set* $E^{*}=\{x\in E:x\notin H(x)\}$ *is open in* *E*;
(ii): *for each* $(x,y)\in E\times E$, $\varsigma(x,y)\subseteq C(x)$ *implies* $\psi(x,y)\subseteq C(x)$;
(iii): *for each* $x\in E$, $\psi(x,x)\nsubseteq C(x)$;
(iv): *for each* $x\in E$, *the set* $\{y\in E:\psi(x,y)\subseteq C(x)\}$ *is convex*;
(v): $\bigcup_{y\in E}\{x\in H^{-1}(y):\psi(x,y)\subseteq C(x)\}= \bigcup_{y\in E}\operatorname{int}\{x\in H^{-1}(y):\psi(x,y)\subseteq C(x)\}$;
(vi): *one of the following conditions holds*:
(vi)_1_: *for each* $N\in{ \mathcal {F}}(E)$, *there exists a nonempty compact convex subset* $E_{N}$ *of* *E* *containing* *N* *such that*
  $$E_{N}\setminus K\subseteq\bigcup_{y\in E_{N}} \operatorname{int}\bigl( \bigl(E^{*}\cap H^{-1}(y) \bigr) \cup \bigl(H^{-1}(y)\cap \bigl\{ x\in E:\psi(x,y) \subseteq C(x) \bigr\} \bigr) \bigr); $$
(vi)_2_: *there exists a point* $y_{0}\in E$ *such that*
  $$E\setminus K\subseteq \operatorname{int}\bigl( \bigl(E^{*}\cap H^{-1}(y_{0}) \bigr)\cup \bigl(H^{-1}(y_{0}) \cap \bigl\{ x\in E:\psi(x,y_{0})\subseteq C(x) \bigr\} \bigr) \bigr). $$

*Then AGVQEP has at least a solution in* *K*.

### Proof

Define two set-valued mappings $L, Q:E\rightrightarrows E$ by

$$L(x)= \bigl\{ y\in E:\varsigma(x,y)\subseteq C(x) \bigr\} \quad \mbox{and}\quad Q(x)= \bigl\{ y \in E:\psi(x,y)\subseteq C(x) \bigr\} ,\quad \forall x\in E. $$

Moreover, let us define two set-valued mappings $F, G:E\rightrightarrows E$ by

$$\begin{aligned} F(x)= \textstyle\begin{cases} H(x)\cap L(x), & \mbox{if } x\notin E^{*}, \\ H(x), & \mbox{if } x\in E^{*}, \end{cases}\displaystyle \end{aligned}$$

and

$$\begin{aligned} G(x)= \textstyle\begin{cases} H(x)\cap Q(x), & \mbox{if } x\notin E^{*}, \\ H(x), & \mbox{if } x\in E^{*}. \end{cases}\displaystyle \end{aligned}$$

By (ii), we have $F(x)\subseteq G(x)$ for every $x\in E$. Since each $Q(x)$ is convex by (iv) and each $H(x)$ is convex by the definition of a Fan-Browder mapping, it follows that $G(x)$ is convex for every $x\in E$.

For each $y\in E$, we have

$$\begin{aligned} G^{-1}(y) =& \bigl\{ x\in E^{*}:y\in H(x) \bigr\} \cup \bigl\{ x\notin E^{*}:y\in H(x) \cap Q(x) \bigr\} \\ =& \bigl(E^{*}\cap H^{-1}(y) \bigr)\cup \bigl( \bigl(E \setminus E^{*} \bigr)\cap \bigl(H^{-1}(y) \cap Q^{-1}(y) \bigr) \bigr) \\ =& \bigl(E^{*}\cap H^{-1}(y) \bigr)\cup \bigl(H^{-1}(y)\cap Q^{-1}(y) \bigr) \\ =& \bigl(E^{*}\cap H^{-1}(y) \bigr)\cup \bigl(H^{-1}(y)\cap \bigl\{ x\in E:\psi(x,y) \subseteq C(x) \bigr\} \bigr). \end{aligned}$$

We claim that $\bigcup_{y\in E}G^{-1}(y)=\bigcup_{y\in E}\operatorname{int}G ^{-1}(y)$. In fact, it is clear that $\bigcup_{y\in E}\operatorname{int}G^{-1}(y) \subseteq\bigcup_{y\in E}G^{-1}(y)$. In order to prove that $\bigcup_{y\in E}G^{-1}(y)\subseteq\bigcup_{y\in E}\operatorname{int}G^{-1}(y)$, we take $x_{0}\in \bigcup_{y\in E}G^{-1}(y)$. Then there exists $y^{*}\in E$ such that

$$x_{0}\in G^{-1} \bigl(y^{*} \bigr)= \bigl(E^{*}\cap H^{-1} \bigl(y^{*} \bigr) \bigr)\cup \bigl(H^{-1} \bigl(y ^{*} \bigr)\cap \bigl\{ x\in E:\psi \bigl(x,y^{*} \bigr)\subseteq C(x) \bigr\} \bigr). $$

If $x_{0}\in E^{*}\cap H^{-1}(y^{*})$, then by (i) and the definition of a Fan-Browder mapping, we have

$$x_{0}\in E^{*}\cap H^{-1} \bigl(y^{*} \bigr)=\operatorname{int}\bigl(E^{*}\cap H^{-1} \bigl(y ^{*} \bigr) \bigr)\subseteq \operatorname{int}G^{-1} \bigl(y^{*} \bigr). $$

We notice that, according to (v) and the definition of *Q*, one has

$$\bigcup_{y\in E} \bigl(H^{-1}(y)\cap Q^{-1}(y) \bigr)=\bigcup_{y\in E} \operatorname{int}\bigl(H^{-1}(y)\cap Q^{-1}(y) \bigr). $$

So, if $x_{0}\in H^{-1}(y^{*})\cap\{x\in E:\psi(x,y^{*})\subseteq C(x)\}=H^{-1}(y^{*})\cap Q^{-1}(y^{*})$, then there exists $\widetilde{y}\in E$ such that

$$x_{0}\in \operatorname{int}\bigl(H^{-1}(\widetilde{y})\cap Q^{-1}(\widetilde{y}) \bigr) \subseteq \operatorname{int}G^{-1}( \widetilde{y}). $$

Combining these two cases, we can conclude that $\bigcup_{y\in E}G ^{-1}(y)\subseteq\bigcup_{y\in E}\operatorname{int}G^{-1}(y)$. Therefore, we have $\bigcup_{y\in E}G^{-1}(y)=\bigcup_{y\in E}\operatorname{int}G^{-1}(y)$.

Now, we show that $x\notin G(x)$ for every $x\in E$. Indeed, if $x\in E^{*}$, then by the definition of $E^{*}$, we have $x\notin H(x)=G(x)$; if $x\notin E^{*}$, then by (iii), $x\notin Q(x)$ and so, $x\notin H(x)\cap Q(x)=G(x)$.

By (vi) and the above fact, we can see that one of the following conditions holds:

- for each $N\in{ \mathcal {F}}(E)$, there exists a nonempty compact convex subset $E_{N}$ of *E* containing *N* such that
  $$\begin{aligned} E_{N}\setminus K \subseteq&\bigcup_{y\in E_{N}} \operatorname{int}\bigl( \bigl(E^{*} \cap H^{-1}(y) \bigr) \cup \bigl(H^{-1}(y)\cap \bigl\{ x\in E:\psi(x,y) \subseteq C(x) \bigr\} \bigr) \bigr) \\ =&\bigcup_{y\in E_{N}}\operatorname{int}G^{-1}(y); \end{aligned}$$
- there exists a point $y_{0}\in E$ such that
  $$\begin{aligned} K \supseteq&E\setminus \operatorname{int}\bigl( \bigl(E^{*}\cap H^{-1}(y_{0}) \bigr)\cup \bigl(H^{-1}(y_{0}) \cap \bigl\{ x\in E:\psi(x,y_{0})\subseteq C(x) \bigr\} \bigr) \bigr) \\ =&\operatorname{cl}\bigl(E\setminus G^{-1}(y_{0}) \bigr). \end{aligned}$$

Therefore, by Lemma 2.5 and Remark 2.3, there exists $\widehat{x} \in K$ such that $G(\widehat{x})=\emptyset$. Since $H(x)\neq\emptyset $ for every $x\in E$, it follows that $\widehat{x}\notin E^{*}$ and $G(\widehat{x})=H(\widehat{x})\cap Q(\widehat{x})=\emptyset$; i.e., $\widehat{x}\in H(\widehat{x})$ and $\psi(\widehat{x},y)\nsubseteq C(\widehat{x})$ for every $y\in H(\widehat{x})$. Thus, the conclusion of Theorem 3.1 holds and the proof is complete. □

### Corollary 3.1

*Let* $K\subseteq E$ *be a nonempty compact set and* *W* *be a nonempty set*. *Let* $\psi:E\times E\rightrightarrows W$, $C:E\rightrightarrows W$ *be two set*-*valued mappings and* $H:E\rightrightarrows E$ *be a Fan*-*Browder mapping*. *Assume that*

(i): *the set* $E^{*}=\{x\in E:x\notin H(x)\}$ *is open in* *E*;
(ii): *for each* $x\in E$, $\psi(x,x)\nsubseteq C(x)$;
(iii): *for each* $x\in E$, *the set* $\{y\in E:\psi(x,y)\subseteq C(x)\}$ *is convex*;
(iv): $\bigcup_{y\in E}\{x\in H^{-1}(y):\psi(x,y)\subseteq C(x)\}= \bigcup_{y\in E}\operatorname{int}\{x\in H^{-1}(y):\psi(x,y)\subseteq C(x)\}$;
(v): *one of the following conditions holds*:
(v)_1_: *for each* $N\in{ \mathcal {F}}(E)$, *there exists a nonempty compact convex subset* $E_{N}$ *of* *E* *containing* *N* *such that*
  $$E_{N}\setminus K\subseteq\bigcup_{y\in E_{N}} \operatorname{int}\bigl( \bigl(E^{*}\cap H^{-1}(y) \bigr) \cup \bigl(H^{-1}(y)\cap \bigl\{ x\in E:\psi(x,y) \subseteq C(x) \bigr\} \bigr) \bigr); $$
(v)_2_: *there exists a point* $y_{0}\in E$ *such that*
  $$E\setminus K\subseteq \operatorname{int}\bigl( \bigl(E^{*}\cap H^{-1}(y_{0}) \bigr)\cup \bigl(H^{-1}(y_{0}) \cap \bigl\{ x\in E:\psi(x,y_{0})\subseteq C(x) \bigr\} \bigr) \bigr). $$

*Then AGVQEP has at least a solution in* *K*.

### Proof

Let $\varsigma=\psi$. It is easy to see that all the conditions of Theorem 3.1 are satisfied. Therefore, it follows from Theorem 3.1 that AGVQEP has at least a solution in *K*. Thus, the result holds and the proof of Corollary 3.1 is complete. □

### Corollary 3.2

*Let* $K\subseteq E$ *be a nonempty compact set and* *W* *be a nonempty set*. *Let* $\psi:E\times E\rightrightarrows W$, $C:E\rightrightarrows W$ *be two set*-*valued mappings and* $H:E\rightrightarrows E$ *be a Fan*-*Browder mapping*. *Assume that*

(i): *the set* $E^{*}=\{x\in E:x\notin H(x)\}$ *is open in* *E*;
(ii): *for each* $x\in E$, $\psi(x,x)\nsubseteq C(x)$;
(iii): *for each* $x\in E$, *the set* $\{y\in E:\psi(x,y)\subseteq C(x)\}$ *is convex*;
(iv): *for each* $y\in E$, *the set* $\{x\in E:\psi(x,y)\subseteq C(x)\}$ *is open in* *E*;
(v): *one of the following conditions holds*:
(v)_1_: *for each* $N\in{ \mathcal {F}}(E)$, *there exists a nonempty compact convex subset* $E_{N}$ *of* *E* *containing* *N* *such that*
  $$E_{N}\setminus K\subseteq\bigcup_{y\in E_{N}} \bigl( \bigl(E^{*}\cap H^{-1}(y) \bigr) \cup \bigl(H^{-1}(y)\cap \bigl\{ x\in E:\psi(x,y)\subseteq C(x) \bigr\} \bigr) \bigr); $$
(v)_2_: *there exists a point* $y_{0}\in E$ *such that*
  $$E\setminus K\subseteq \bigl( \bigl(E^{*}\cap H^{-1}(y_{0}) \bigr)\cup \bigl(H^{-1}(y _{0})\cap \bigl\{ x\in E: \psi(x,y_{0})\subseteq C(x) \bigr\} \bigr) \bigr). $$

*Then AGVQEP has at least a solution in* *K*.

### Proof

By (iv), (v), and the fact that $H^{-1}(y)$ is open in *E* for every $y\in E$, we can see that (iv) and (v) of Corollary 3.1 hold. Therefore, by Corollary 3.1, AGVQEP has at least a solution in *K*. This completes the proof. □

### Remark 3.3

Corollary 3.2 extends Theorem 3.1 of Batista et al. [25] in the following aspects: (a) concerns the more general abstract generalized vector quasi-equilibrium problems instead of the generalized vector equilibrium problems; (b) from one coercivity condition to two alternative coercivity conditions; (c) since *W* in Corollary 3.2 does not need to be a real Hausdorff topological vector space, it is not required for each $C(x)$ to be a closed and convex cone; (d) (iii) is weaker than h3 of Theorem 3.1 of Batista et al. [25]; (e) by the fact that *W* in Corollary 3.2 may be any nonempty set without topological structure, we adopt the assumption that the set $\{x\in E:\psi(x,y) \subseteq C(x)\}$ is open in *E* for every $y\in E$, which is weaker than h2 of Theorem 3.1 of Batista et al. [25]. In addition, the proof of Corollary 3.2 originates from the existence of maximal elements in noncompact Hadamard manifolds, while the authors of [25] used the KKM property to prove their result. Therefore, the proof technique of Corollary 3.2 is different from that of Theorem 3.1 of Batista et al. [25].

By using Corollary 3.2, we can prove the existence of an equilibrium for the generalized water market game model under the condition that there are a river structure, water balances, and heterogeneous water users via a water delivery infrastructure. We would like to point out that our convex and continuous conditions are weaker than the corresponding conditions in Proposition 2.1 due to Ansink and Houba [41].

### Corollary 3.3

*Let* $K\subseteq E$ *be a nonempty compact set*, *W* *be a nonempty set*, *and let* $C:E\rightrightarrows W$, $\psi:E\times E\rightrightarrows W$ *be two set*-*valued mappings*. *Assume that*

(i): *for each* $x\in E$, $\psi(x,x)\nsubseteq C(x)$;
(ii): *for each* $x\in E$, *the set* $\{y\in E:\psi(x,y)\subseteq C(x)\}$ *is convex*;
(iii): *for each* $y\in E$, *the set* $\{x\in E:\psi(x,y)\subseteq C(x)\}$ *is open in* *E*;
(iv): *one of the following conditions holds*:
(iv)_1_: *for each* $N\in{ \mathcal {F}}(E)$, *there exists a nonempty compact convex subset* $E_{N}$ *of* *E* *containing* *N* *such that* $E_{N}\setminus K\subseteq\bigcup_{y\in E_{N}}\{x\in E:\psi(x,y) \subseteq C(x)\}$;
(iv)_2_: *there exists a point* $y_{0}\in E$ *such that* $E\setminus K\subseteq\{x\in E:\psi(x,y_{0})\subseteq C(x)\}$.

*Then AVQEP has at least a solution in* *K*.

### Proof

The conclusion of Corollary 3.3 follows from Corollary 3.2 by letting $H(x)\equiv E$ for every $x\in E$. This completes the proof. □

### Remark 3.4

Let us give the following items:

(1) If *W* is a real Hausdorff topological vector space and $\{C(x):y\in E\}$ is a family of nonempty convex cones, then (iv) of Theorem 3.1, (iii) of Corollaries 3.1-3.2, and (ii) of Corollary 3.3 can be replaced by one of the following conditions:

- for each $x\in E$, $\psi(x,\cdot)$ is convex with respect to $C(x)$;
- for each $x\in E$, $\psi(x,\cdot)$ is quasiconvex-like with respect to $C(x)$.

In fact, we consider the first assumption. Let $x\in E$ be any given. In order to prove that the set $\{y\in E:\psi(x,y)\subseteq C(x) \}$ is convex, we assume that $y_{1}, y_{2}\in\{y\in E:\psi(x,y) \subseteq C(x)\}$ and $t\in[0,1]$. Applying this assumption and the fact that each $C(x)$ is a convex cone, we have

$$\begin{aligned} \psi \bigl(x,\operatorname{exp}_{y_{1}}t\operatorname{exp}_{y_{1}}^{-1}y_{2} \bigr) \subseteq&t \psi(x,y_{1})+(1-t)\psi(x,y_{2})+C(x) \\ \subseteq&tC(x)+(1-t)C(x)+C(x) \\ \subseteq&C(x), \end{aligned}$$

which implies that $\operatorname{exp}_{y_{1}}t\operatorname{exp}_{y_{1}}^{-1}y_{2} \in\{y\in E:\psi(x,y)\subseteq C(x)\}$ for every $t\in[0,1]$ and so, the set $\{y\in E:\psi(x,y)\subseteq C(x)\}$ is convex for every $x\in E$. Now, we prove that the desired conclusion holds under the condition that the second assumption is satisfied. Indeed, let $x\in E$ be fixed and then let $y_{1}, y_{2}\in\{y\in E:\psi(x,y) \subseteq C(x)\}$ and $t\in[0,1]$ be any given. By the definition of a quasiconvex-like mapping, we have

$$\psi \bigl(x,\operatorname{exp}_{y_{1}}t\operatorname{exp}_{y_{1}}^{-1}y_{2} \bigr)\subseteq \psi(x,y_{1})+C(x), $$

or

$$\psi \bigl(x,\operatorname{exp}_{y_{1}}t\operatorname{exp}_{y_{1}}^{-1}y_{2} \bigr)\subseteq \psi(x,y_{2})+C(x). $$

Since $y_{1}, y_{2}\in\{y\in E:\psi(x,y)\subseteq C(x)\}$, we have $\psi(x,y_{1})\subseteq C(x)$ and $\psi(x,y_{2})\subseteq C(x)$. Therefore, given $C(x)+C(x)\subseteq C(x)$, which is obtained by using the property of a convex cone, it follows from the above formulas that $\psi(x,\operatorname{exp}_{y_{1}}t\operatorname{exp}_{y_{1}}^{-1}y_{2})\subseteq C(x)$, and this shows that the set $\{y\in E:\psi(x,y)\subseteq C(x)\}$ is convex for every $x\in E$.

(2) If *W* is a Hausdorff topological space, then (iv) of Corollary 3.2 and (iii) of Corollary 3.3 can be replaced by the following conditions:

- for each $y\in E$, $\psi(\cdot,y)$ is upper semicontinuous on *E* with compact values;
- the graph $G_{r}(C)$ of *C*; i.e., $\{(x,w)\in E\times W:w\in C(x)\}$ is an open set in $E\times W$.

Indeed, it suffices to prove that the set $\{x\in E:\psi(x,y)\nsubseteq C(x)\}$ is closed in *E* for every $y\in E$. Let $\{x_{ \alpha}\}$ be a net in $\{x\in E:\psi(x,y)\nsubseteq C(x)\}$ such that $x_{\alpha}\rightarrow x_{0}$. Since $\psi(x_{\alpha},y) \nsubseteq C(x_{\alpha})$, there exists $z_{\alpha}\in\psi(x _{\alpha},y)$ such that $z_{\alpha}\notin C(x_{\alpha})$. Hence, we have $z_{\alpha}\in W\setminus C(x_{\alpha})$. By the upper semicontinuity and compact values of *ψ* on *E*, it follows from Proposition 1 in [42] that there exists a subnet of $\{z_{\alpha}\}$ with limit $z_{0}$ and $z_{0}\in\psi(x_{0},y)$. Without loss of generality, let us assume that $z_{\alpha}\rightarrow z_{0}\in\psi(x_{0},y)$. On the other hand, since the graph $G_{r}(C)$ of *C* is an open set in $E\times W$, the set-valued mapping $x\rightrightarrows W\setminus C(x)$ has a closed graph in $E\times W$. It follows that $z_{0}\in W\setminus C(x_{0})$ and so, $z_{0}\notin C(x_{0})$. Thus, $x_{0}\in\{x\in E: \psi(x,y)\nsubseteq C(x)\}$, which implies that the set $\{x\in E:\psi(x,y)\nsubseteq C(x)\}$ is closed in *E* for every $y\in E$. Therefore, the set $\{x\in E:\psi(x,y)\subseteq C(x)\}$ is open in *E* for every $y\in E$.

(3) If (iv)_2_ of Corollary 3.3 holds, then the solution set of AVQEP is a nonempty compact set, which can be written as follows:

$$\bigcap_{y\in E} \bigl\{ x\in E:\psi(x,y)\nsubseteq C(x) \bigr\} . $$

In fact, by the conclusion of Corollary 3.3, we can see that the above set is nonempty. Furthermore, it follows from (iv)_2_ of Corollary 3.3 that

$$\begin{aligned} \emptyset\neq\bigcap_{y\in E} \bigl\{ x\in E:\psi(x,y) \nsubseteq C(x) \bigr\} \subseteq& \bigl\{ x\in E:\psi(x,y_{0}) \subseteq C(x) \bigr\} \subseteq K. \end{aligned}$$

Together with (iii) of Corollary 3.3, we can see that the solution set of AVQEP is a nonempty closed subset of *K*. Therefore, the solution set of AVQEP is a nonempty compact set.

If $W=\mathbb{R}$, $C(x)\equiv(-\infty,0)$ for every $x\in E$ and $F=f$, where $f:E\times E\rightarrow\mathbb{R}$ is a bifunction, then Corollaries 3.2 and 3.3 reduce to the following existence results of solutions to GSEP and SEP, respectively.

### Corollary 3.4

*Let* $K\subseteq E$ *be a nonempty compact set*, $H:E\rightrightarrows E$ *be a Fan*-*Browder mapping*, *and* $f:E\times E\rightarrow\mathbb{R}$ *be a bifunction*. *Assume that*

(i): *the set* $E^{*}=\{x\in E:x\notin H(x)\}$ *is open in* *E*;
(ii): *for each* $x\in E$, $f(x,x)\geq0$;
(iii): *for each* $x\in E$, *the set* $\{y\in E:f(x,y)< 0\}$ *is convex*;
(iv): *for each* $y\in E$, *the set* $\{x\in E:f(x,y)\geq0\}$ *is closed in* *E*;
(v): *one of the following conditions holds*:
(v)_1_: *for each* $N\in{ \mathcal {F}}(E)$, *there exists a nonempty compact convex subset* $E_{N}$ *of* *E* *containing* *N* *such that*
  $$E_{N}\setminus K\subseteq\bigcup_{y\in E_{N}} \bigl( \bigl(E^{*}\cap H^{-1}(y) \bigr) \cup \bigl(H^{-1}(y)\cap \bigl\{ x\in E:f(x,y)< 0 \bigr\} \bigr) \bigr); $$
(v)_2_: *there exists a point* $y_{0}\in E$ *such that*
  $$E\setminus K\subseteq \bigl( \bigl(E^{*}\cap H^{-1}(y_{0}) \bigr)\cup \bigl(H^{-1}(y _{0})\cap \bigl\{ x\in E:f(x,y_{0})< 0 \bigr\} \bigr) \bigr). $$

*Then GSEP has at least a solution in* *K*.

### Corollary 3.5

*Let* $K\subseteq E$ *be a nonempty compact set and* $f:E\times E\rightarrow\mathbb{R}$ *be a bifunction*. *Assume that*

(i): *for each* $x\in E$, $f(x,x)\geq0$;
(ii): *for each* $x\in E$, $\{y\in E:f(x,y)<0\}$ *is convex*;
(iii): *for each* $y\in E$, *the set* $\{x\in E:f(x,y)\geq0\}$ *is closed in* *E*;
(iv): *one of the following conditions holds*:
(iv)_1_: *for each* $N\in{ \mathcal {F}}(E)$, *there exists a nonempty compact convex subset* $E_{N}$ *of* *E* *containing* *N* *such that* $E_{N}\setminus K\subseteq\bigcup_{y\in E_{N}}\{x\in E:f(x,y)<0\}$;
(iv)_2_: *there exists a point* $y_{0}\in E$ *such that* $E\setminus K\subseteq\{x\in E:f(x,y_{0})<0\}$.

*Then SEP has at least a solution in* *K*.

### Remark 3.5

Corollary 3.5 improves Theorem 3.2 of Colao et al. [21] because there are two alternative coercivity conditions in Corollary 3.5, while there is only one coercivity condition in Theorem 3.2 of Colao et al. [21]. Furthermore, the coercivity condition (iv)_2_ of Corollary 3.5 is weaker than the coercivity condition (iv) of Theorem 3.2 of Colao et al. [21]. To see this, we can consider *K* in Theorem 3.2 of Colao et al. [21] as a Hadamard submanifold, and then let $L'=L\cap K$, which is a nonempty compact subset of *K*. Then it follows from $K\setminus L=K\setminus L'$ and $y_{0}\in L\cap K\subseteq K$ that (iv) of Theorem 3.2 of Colao et al. [21] implies (iv)_2_ of Corollary 3.5.

As an application of Corollary 3.5, we have the following perturbed saddle point theorem in noncompact Hadamard manifolds.

### Theorem 3.2

*Let* $K_{1}, K_{2}\subseteq E$ *be two nonempty compact sets and* $f, g:E\times E\rightarrow\mathbb{R}$ *be two bifunctions*. *Assume that*

(i): *for each* $x\in E$, $f(x,x)-g(x,x)=0$;
(ii): *for each* $x\in E$, $\{y\in E:f(x,y)< g(x,y)\}$ *is convex*;
(iii): *for each* $y\in E$, $\{x\in E:f(x,y)>g(x,y)\}$ *is convex*;
(iv): *the bifunction* $E\times E\ni(x,y)\mapsto f(x,y)-g(x,y)$ *is continuous*;
(v): *one of the following conditions holds*:
(v)_1_: *for each* $N\in{ \mathcal {F}}(E)$, *there exist two nonempty compact convex subsets* $E_{N}$, $\widetilde{E}_{N}$ *of* *E* *containing* *N* *such that* $E_{N}\setminus K_{1}\subseteq \bigcup_{y\in E_{N}}\{x\in E:f(x,y)< g(x,y)\}$ *and* $\widetilde{E}_{N} \setminus K_{2}\subseteq\bigcup_{x\in\widetilde{E}_{N}}\{y\in E:f(x,y)>g(x,y) \}$;
(v)_2_: *there exist two points* $y_{0}, x_{0}\in E$ *such that* $E\setminus K_{1}\subseteq\{x\in E:f(x,y_{0})< g(x,y_{0})\}$ *and* $E\setminus K_{2}\subseteq\{y\in E:f(x_{0},y)>g(x_{0},y)\}$.

*Then* *f* *has a perturbed saddle point* $(\widehat{x},\widehat{y}) \in K_{1}\times K_{2}$; *i*.*e*.,

$$f(x,\widehat{y})+ \bigl[g(\widehat{x},\widehat{y})-g(x,\widehat{y}) \bigr]\leq f( \widehat{x},\widehat{y})\leq f(\widehat{x},y)+ \bigl[g(\widehat{x}, \widehat{y})-g( \widehat{x},y) \bigr],\quad \forall (x,y)\in E\times E. $$

*In particular*, $\inf_{y\in E}\sup_{x\in E}[f(x,y)-g(x,y)]=\sup_{x \in E}\inf_{y\in E}[f(x,y)-g(x,y)]$.

### Proof

Define a bifunction $h_{1}:E\times E\rightarrow \mathbb{R}$ by $h_{1}(x,y)=f(x,y)-g(x,y)$ for every $(x,y)\in E\times E$. By (i), (ii), (iv) and the first parts of (v)_1_ and (v)_2_, we can see that all the conditions of Corollary 3.5 are satisfied. Thus, by Corollary 3.5, there exists $\widehat{x}\in K_{1}$ such that $h_{1}(\widehat{x},y)\geq0$ for every $y\in E$. Define a bifunction $h_{2}:E\times E\rightarrow\mathbb{R}$ by $h_{2}(y,x)=g(x,y)-f(x,y)$ for every $(y,x)\in E\times E$. Then it follows from (i), (iii), (iv), and the second parts of (v)_1_ and (v)_2_ that all the hypotheses of Corollary 3.5 are fulfilled. Thus, by Corollary 3.5 again, there exists $\widehat{y}\in K_{2}$ such that $h_{2}(\widehat{y},x)\geq0$ for every $x\in E$. Therefore, we have $f(\widehat{x},\widehat{y})-g( \widehat{x},\widehat{y})=0$ and

$$f(x,\widehat{y})-g(x,\widehat{y})\leq0=f(\widehat{x},\widehat{y})-g( \widehat{x},\widehat{y})\leq f(\widehat{x},y)-g(\widehat{x},y),\quad \forall (x,y) \in E \times E. $$

It follows from the above inequality that

$$f(x,\widehat{y})+ \bigl[g(\widehat{x},\widehat{y})-g(x,\widehat{y}) \bigr]\leq f( \widehat{x},\widehat{y})\leq f(\widehat{x},y)+ \bigl[g(\widehat{x}, \widehat{y})-g( \widehat{x},y) \bigr], \quad \forall (x,y)\in E\times E, $$

and

$$\inf_{y\in E}\sup_{x\in E} \bigl[f(x,y)-g(x,y) \bigr]\leq\sup_{x\in E}\inf_{y \in E} \bigl[f(x,y)-g(x,y) \bigr]. $$

Since $\inf_{y\in E}\sup_{x\in E}[f(x,y)-g(x,y)]\geq\sup_{x\in E} \inf_{y\in E}[f(x,y)-g(x,y)]$ is always true, we get

$$\inf_{y\in E}\sup_{x\in E} \bigl[f(x,y)-g(x,y) \bigr]=\sup_{x\in E}\inf_{y\in E} \bigl[f(x,y)-g(x,y) \bigr]. $$

This completes the proof. □

By setting $g(x,y)\equiv0$ for every $(x,y)\in E\times E$, we obtain the following saddle point theorem from Theorem 3.2.

### Theorem 3.3

*Let* $K_{1}, K_{2}\subseteq E$ *be two nonempty compact sets and* $f:E\times E\rightarrow\mathbb{R}$ *be a bifunction*. *Assume that*

(i): *for each* $x\in E$, $f(x,x)=0$;
(ii): *for each* $x\in E$, $\{y\in E:f(x,y)<0\}$ *is convex*;
(iii): *for each* $y\in E$, $\{x\in E:f(x,y)>0\}$ *is convex*;
(iv): *the bifunction* $E\times E\ni(x,y)\mapsto f(x,y)$ *is continuous*;
(v): *one of the following conditions holds*:
(v)_1_: *for each* $N\in{ \mathcal {F}}(E)$, *there exist two nonempty compact convex subsets* $E_{N}$, $\widetilde{E}_{N}$ *of* *E* *containing* *N* *such that* $E_{N}\setminus K_{1}\subseteq \bigcup_{y\in E_{N}}\{x\in E:f(x,y)<0\}$ *and* $\widetilde{E}_{N}\setminus K_{2}\subseteq\bigcup_{x\in\widetilde{E}_{N}}\{y\in E:f(x,y)>0\}$;
(v)_2_: *there exist two points* $y_{0}, x_{0}\in E$ *such that* $E\setminus K_{1}\subseteq\{x\in E:f(x,y_{0})<0\}$ *and* $E\setminus K _{2}\subseteq\{y\in E:f(x_{0},y)>0\}$.

*Then* *f* *has a saddle point* $(\widehat{x},\widehat{y})\in K_{1} \times K_{2}$; *i*.*e*.,

$$f(x,\widehat{y})\leq f(\widehat{x},\widehat{y})\leq f(\widehat{x},y),x\quad \forall (x,y)\in E\times E. $$

*In particular*, $\inf_{y\in E}\sup_{x\in E}f(x,y)=\sup_{x\in E} \inf_{y\in E}f(x,y)$.

## Weakly mixed variational inequality problem

In this section, inspired by the idea due to Colao et al. [21], we introduce WMVIP in Hadamard manifolds and present a sufficient condition for the existence of solutions to WMVIP. Let $H:E\rightrightarrows E$ be a set-valued mapping, $\sigma:E\rightarrow TE$ be a vector field, and $\varphi:E\rightarrow\mathbb{R}$ be a real-valued function. We consider the following WMVIP: find $\widehat{x}\in E$ such that

$$\widehat{x}\in H(\widehat{x}) \quad \mbox{and}\quad \bigl\langle \sigma( \widehat{x}), \operatorname{exp}^{-1}_{\widehat{x}}y \bigr\rangle +\varphi(y)- \varphi( \widehat{x})\geq0,\quad \forall y\in H(\widehat{x}). $$

### Remark 4.1

WMVIP contains the mixed variational inequality problem (for short, MVIP) considered by Colao et al. [21]. In fact, let $H(x)\equiv E$ for every $x\in E$. Then WMVIP collapses to MVIP in [21]. Furthermore, if $\varphi(y)\equiv0$ for every $y\in E$, then WMVIP coincides with the following variational inequality problem.

$$\mbox{Find } \widehat{x}\in E \mbox{ such that } \bigl\langle \sigma( \widehat{x}), \operatorname{exp}^{-1}_{\widehat{x}}y \bigr\rangle \geq0,\quad \forall y\in E, $$

which was introduced and studied by Németh [26].

By Corollary 3.4, we have the following existence theorem of solutions to WMVIP in noncompact Hadamard manifolds.

### Theorem 4.1

*Let* $K\subseteq E$ *be a nonempty compact set*, $H:E\rightrightarrows E$ *be a Fan*-*Browder mapping*, $\varphi:E\rightarrow \mathbb{R}$ *be a lower semicontinuous function*, *and* $\sigma:E\rightarrow TE$ *be a continuous vector field*. *Assume that*

(i): *the set* $E^{*}=\{x\in E:x\notin H(x)\}$ *is open in* *E*;
(ii): *for each* $x\in E$, *the function* $E\ni y\mapsto\langle \sigma(x),\operatorname{exp}^{-1}_{x}y\rangle+\varphi(y)$ *is convex*;
(iii): *one of the following conditions holds*:
(iii)_1_: *for each* $N\in{ \mathcal {F}}(E)$, *there exists a nonempty compact convex subset* $E_{N}$ *of* *E* *containing* *N* *such that*
  $$E_{N}\setminus K\subseteq\bigcup_{y\in E_{N}} \bigl( \bigl(E^{*}\cap H^{-1}(y) \bigr) \cup \bigl(H^{-1}(y)\cap \bigl\{ x\in E: \bigl\langle \sigma(x),\operatorname{exp}^{-1} _{x}y \bigr\rangle < \varphi(x)- \varphi(y) \bigr\} \bigr) \bigr); $$
(iii)_2_: *there exists a point* $y_{0}\in E$ *such that*
  $$E\setminus K\subseteq \bigl( \bigl(E^{*}\cap H^{-1}(y_{0}) \bigr)\cup \bigl(H^{-1}(y _{0})\cap \bigl\{ x\in E: \bigl\langle \sigma(x), \operatorname{exp}^{-1}_{x}y_{0} \bigr\rangle < \varphi(x)- \varphi(y_{0}) \bigr\} \bigr) \bigr). $$

*Then WMVIP has at least a solution in* *K*.

### Proof

Define a bifunction $f:E\times E\rightarrow\mathbb{R}$ by

$$f(x,y)= \bigl\langle \sigma(x),\operatorname{exp}^{-1}_{x}y \bigr\rangle + \varphi(y)- \varphi(x),\quad \forall (x,y)\in E\times E. $$

It is clear that $f(x,x)\geq0$ for every $x\in E$. Thus, (ii) of Corollary 3.4 is satisfied. By Lemma 2.2 and the continuous properties of *σ* and *φ*, one can see that the function $E\ni x \mapsto f(x,y)$ is upper semicontinuous. Therefore, the set $\{x\in E:f(x,y)\geq0\}$ is closed in *E* for every $x\in E$, which implies that (iv) of Corollary 3.4 holds. Moreover, it follows from (iii) and the definition of *f* that one of the following conditions holds:

- for each $N\in{ \mathcal {F}}(E)$, there exists a nonempty compact convex subset $E_{N}$ of *E* containing *N* such that
  $$E_{N}\setminus K\subseteq\bigcup_{y\in E_{N}} \bigl( \bigl(E^{*}\cap H^{-1}(y) \bigr) \cup \bigl(H^{-1}(y)\cap \bigl\{ x\in E:f(x,y)< 0 \bigr\} \bigr) \bigr); $$
- there exists a point $y_{0}\in E$ such that
  $$E\setminus K\subseteq \bigl( \bigl(E^{*}\cap H^{-1}(y_{0}) \bigr)\cup \bigl(H^{-1}(y _{0})\cap \bigl\{ x\in E:f(x,y_{0})< 0 \bigr\} \bigr) \bigr). $$

Thus, (v) of Corollary 3.4 is satisfied. Now, in order to show that (iii) of Corollary 3.4 holds, we are ready to prove that $\{y\in E:f(x,y)<0 \}$ is convex for every $x\in E$. Indeed, let $x\in E$ be fixed and let $y_{1}, y_{2}\in\{y\in E:f(x,y)=\langle\sigma(x),\operatorname{exp}^{-1} _{x}y\rangle+\varphi(y)-\varphi(x)<0\}$, $t\in[0,1]$. By (ii), we get

$$\begin{aligned} f \bigl(x,\operatorname{exp}_{y_{1}}t\operatorname{exp}_{y_{1}}^{-1}y_{2} \bigr) =& \bigl\langle \sigma (x),\operatorname{exp}^{-1}_{x} \bigl( \operatorname{exp}_{y_{1}}t\operatorname{exp}_{y_{1}}^{-1}y _{2} \bigr) \bigr\rangle +\varphi \bigl(\operatorname{exp}_{y_{1}}t\operatorname{exp}_{y_{1}}^{-1}y _{2} \bigr)-\varphi(x) \\ \leq&t \bigl( \bigl\langle \sigma(x),\operatorname{exp}^{-1}_{x}y_{1} \bigr\rangle +\varphi (y_{1}) \bigr) \\ &{}+(1-t) \bigl( \bigl\langle \sigma(x),\operatorname{exp}^{-1}_{x}y_{2} \bigr\rangle +\varphi (y_{2}) \bigr)-\varphi(x) \\ =&t \bigl( \bigl\langle \sigma(x),\operatorname{exp}^{-1}_{x}y_{1} \bigr\rangle +\varphi(y _{1})-\varphi(x) \bigr) \\ &{}+(1-t) \bigl( \bigl\langle \sigma(x),\operatorname{exp}^{-1}_{x}y_{2} \bigr\rangle +\varphi (y_{2})-\varphi(x) \bigr) < 0, \end{aligned}$$

which implies that the set $\{y\in E:f(x,y)<0\}$ is convex for every $x\in E$. As a consequence of Corollary 3.4, there exists a point $\widehat{x}\in K$ such that $\widehat{x}\in H(\widehat{x}) \mbox{ and } f(\widehat{x},y)=\langle\sigma(\widehat{x}),\operatorname{exp}^{-1}_{\widehat{x}}y\rangle+\varphi(y)-\varphi(\widehat{x})\geq0 \mbox{ for every } y\in H(\widehat{x})$; that is, WMVIP has at least a solution in *K*. This completes the proof. □

### Remark 4.2

The following two conditions are stronger than (iii)_1_ and (iii)_2_ of Theorem 4.1, respectively.

$(\mathrm{iii})^{\prime}_{1}$
For each $N\in{ \mathcal {F}}(E)$, there exists a nonempty compact convex subset $E_{N}$ of *E* containing *N* such that for all $x\in E_{N}\setminus K$, there exists $y\in E_{N}$ such that $y\in H(x)$ and $\langle\sigma(x),\operatorname{exp}^{-1}_{x}y\rangle< \varphi(x)-\varphi(y)$;
$(\mathrm{iii})^{\prime}_{2}$
There exists a point $y_{0}\in E$ such that $y_{0}\in H(x)$ and $\langle\sigma(x),\operatorname{exp}^{-1}_{x}y_{0} \rangle<\varphi(x)-\varphi(y_{0})$ for every $x\in E\setminus K$.

If $H(x)\equiv E$ for every $x\in E$, then we can obtain the following corollary from Theorem 4.1.

### Corollary 4.1

*Let* $K\subseteq E$ *be a nonempty compact set*, $\varphi:E\rightarrow\mathbb{R}$ *be a lower semicontinuous function*, *and* $\sigma:E\rightarrow TE$ *be a continuous vector field*. *Assume that*

(i): *for each* $x\in E$, *the function* $E\ni y\mapsto\langle \sigma(x),\operatorname{exp}^{-1}_{x}y\rangle+\varphi(y)$ *is convex*;
(ii): *one of the following conditions holds*:
(ii)_1_: *for each* $N\in{ \mathcal {F}}(E)$, *there exists a nonempty compact convex subset* $E_{N}$ *of* *E* *containing* *N* *such that* $E_{N}\setminus K\subseteq\bigcup_{y\in E_{N}}\{x\in E:\langle \sigma(x),\operatorname{exp}^{-1}_{x}y\rangle<\varphi(x)-\varphi(y)\}$;
(ii)_2_: *there exists* $y_{0}\in E$ *such that* $E\setminus K \subseteq\{x\in E:\langle\sigma(x),\operatorname{exp}^{-1}_{x}y_{0}\rangle <\varphi(x)-\varphi(y_{0})\}$.

*Then MVIP has at least a solution in* *K*.

### Remark 4.3

Corollary 4.1 extends Theorem 3.5 of Colao et al. [21] in the following aspects: (a) from one coercivity condition to two coercivity conditions; (b) the coercivity condition (ii)_2_ of Corollary 4.1 is weaker than the coercivity condition (C) of Theorem 3.5 of Colao et al. [21] in view of the same argument as in Remark 3.5; (c) by Lemma 2.3 and the proof of Theorem 3.5 of Colao et al. [21], we can see that the sectional curvature of the Hadamard manifold in Theorem 3.5 of Colao et al. [21] is identically zero, while it is not required for the sectional curvature of the Hadamard manifold in Corollary 4.1 to be identically zero; (d) the convexity of the function *f* in Theorem 3.5 of Colao et al. [21] is dropped.

### Corollary 4.2

*Let* $\varphi:E\rightarrow\mathbb{R}$ *be a convex lower semicontinuous function and* $\sigma:E\rightarrow TE$ *be a continuous vector field such that the function* $E\ni y\mapsto \langle\sigma(x),\operatorname{exp}^{-1}_{x}y\rangle+\varphi(y)$ *is convex for every* $x\in E$. *Suppose that one of the following conditions holds*:

(i)_1_: *E* *is compact*;
(i)_2_: *there exists* $y_{0}\in E$ *such that*, *for each* $u\in T_{y_{0}}E$, *the following condition holds*:
  $$\lim_{d(y_{0},x)\rightarrow+\infty}\frac{\langle\sigma(y_{0}), \operatorname{exp}_{y_{0}}^{-1}x\rangle+\langle\sigma(x),\operatorname{exp}_{x} ^{-1}y_{0}\rangle}{d(y_{0},x)}< - \bigl( \bigl\Vert \sigma(y_{0}) \bigr\Vert +\Vert u\Vert \bigr). $$

*Then MVIP has at least a solution*.

### Proof

Suppose that (i)_1_ holds. For each $N\in{ \mathcal {F}}(E)$, let $E_{N}=E=K$. Thus, both (ii)_1_ and (ii)_2_ of Corollary 4.1 are satisfied automatically. Therefore, MVIP has at least a solution in *E*. Now, we suppose that (i)_2_ is satisfied. Let $y_{0}\in E$ satisfy (i)_2_. Since *φ* is convex, it follows from Lemma 2.4 that $D(\partial\varphi)=E$. Thus, we may choose $u_{0}\in\partial\varphi(y_{0})\subseteq T_{y_{0}}E$; i.e., the subdifferential of *φ* at $y_{0}$. For each $x\in E$, by the generalized Cauchy-Schwarz inequality, we have

$$\begin{aligned} \varphi(y_{0})-\varphi(x) \leq&- \bigl\langle u_{0}, \operatorname{exp}^{-1}_{y_{0}}x \bigr\rangle \leq \Vert u_{0}\Vert \bigl\Vert \operatorname{exp}^{-1}_{y_{0}}x \bigr\Vert =\Vert u_{0}\Vert d(y_{0},x). \end{aligned}$$

It follows from the above relation that

$$- \bigl\langle \sigma(y_{0}),\operatorname{exp}_{y_{0}}^{-1}x \bigr\rangle +\varphi(y_{0})- \varphi(x)\leq \bigl( \bigl\Vert \sigma(y_{0}) \bigr\Vert +\Vert u_{0}\Vert \bigr)d(y_{0},x), \quad \forall x\in E. $$

By (i)_2_, there exists $\lambda\in\mathbb{R}$ such that

$$\lim_{d(y_{0},x)\rightarrow+\infty}\frac{\langle\sigma(y_{0}), \operatorname{exp}_{y_{0}}^{-1}x\rangle+\langle\sigma(x),\operatorname{exp}_{x} ^{-1}y_{0}\rangle}{d(y_{0},x)}< -\lambda< - \bigl( \bigl\Vert \sigma (y_{0}) \bigr\Vert +\Vert u_{0}\Vert \bigr). $$

We can choose $r>0$ large enough such that, for each $x\notin B(y _{0},r)=\{x\in E:d(x,y_{0})\leq r\}$, we have $\langle\sigma(y_{0}), \operatorname{exp}_{y_{0}}^{-1}x\rangle+\langle\sigma(x),\operatorname{exp}_{x} ^{-1}y_{0}\rangle\leq-\lambda d(y_{0},x)$. Then, for each $x\in E\setminus B(y_{0},r)$, we have the following:

$$\begin{aligned} \bigl\langle \sigma(x),\operatorname{exp}_{x}^{-1}y_{0} \bigr\rangle +\varphi(y_{0})- \varphi(x) \leq&- \bigl\langle \sigma(y_{0}),\operatorname{exp}_{y_{0}}^{-1}x \bigr\rangle - \lambda d(y_{0},x)+\varphi(y_{0})-\varphi(x) \\ \leq& \bigl( \bigl\Vert \sigma(y_{0}) \bigr\Vert +\Vert u_{0}\Vert -\lambda \bigr)d(y_{0},x) \\ < &0. \end{aligned}$$

Let $K=B(y_{0},r)$. Since *K* is a bounded closed set in *E*, it follows from the Hopf-Rinow theorem that *K* is a compact subset of *E*. Thus, by the above inequality, one can see that (ii)_2_ of Corollary 4.1 holds. Therefore, by Corollary 4.1, MVIP has at least a solution. This completes the proof. □

### Remark 4.4

Corollary 4.2 generalizes Corollary 3.6 of Colao et al. [21] in the following two aspects: (a) from the Hadamard manifold with its sectional curvature being identically zero to the Hadamard manifold with its sectional curvature being nonpositive. This fact can be deduced from Lemma 2.3; (b) (ii)_2_ of Corollary 4.2 is weaker than (ii) of Corollary 3.6 of Colao et al. [21].

## Conclusions

In this paper, we introduce and study AGVQEP in noncompact Hadamard manifolds. By means of a maximal element theorem, we establish an existence theorem for a solution to AGVQEP in noncompact Hadamard manifolds. Moreover, we provide applications to AVQEP, GSEP, SEP, and the perturbed saddle point problem. Finally, WMVIP in noncompact Hadamard manifolds is introduced, and by applying our results, a weakly mixed variational inequality and two mixed variational inequalities are established.
